# Methodological considerations in establishing and maintaining longitudinal health workforce studies: Lessons learned from the WiSDOM cohort in South Africa

**DOI:** 10.1080/16549716.2021.1996688

**Published:** 2021-12-20

**Authors:** Laetitia C. Rispel, Prudence Ditlopo, Janine White, Duane Blaauw

**Affiliations:** a Centre for Health Policy & South African Research Chairs Initiative (SARChI), School of Public Health, Faculty of Health Sciences, University of the Witwatersrand, Johannesburg, South Africa; b Centre for Health Policy, School of Public Health, Faculty of Health Sciences, University of the Witwatersrand, Johannesburg, South Africa; c School of Public Health, Faculty of Health Sciences, University of the Witwatersrand, Johannesburg, South Africa

**Keywords:** Cohort, health system, human resources for health, labour market, South Africa

## Abstract

**Background:**

Health workforce cohort studies are uncommon in low-and middle-income countries (LMICs), especially those in sub-Saharan Africa.

**Objective:**

Describe the methodology and lessons learned from establishing and maintaining the WiSDOM (Wits longitudinal Study to Determine the Operation of the labour Market among its health professional graduates) health professional cohort study in South Africa.

**Methods:**

WiSDOM is a prospective longitudinal cohort study that commenced in 2017. The cohort focuses on the eight professional groups of clinical associates, dentists, doctors, nurses, occupational therapists, oral hygienists, pharmacists and physiotherapists. Annual, electronic follow-up surveys have been conducted in 2018, 2019 and 2020 with informed consent. Key steps in establishing the WiSDOM cohort include consultation, communication and marketing, stakeholder feedback, resources and infrastructure. Retention strategies consist of an electronic database, detailed cohort contact information, cohort engagement, communication and feedback, short survey tools, and appropriate incentives.

**Results:**

We obtained an overall response rate of 89.5% at baseline in 2017, 79.6% in 2018, 68.3% in 2019 and 72.8% in 2020. The largest decline in response rates is for medical doctors: 66.0% response rate in 2018, 53.2% in 2019 and 58.2% in 2020. However, for each of the three follow-up surveys, we have obtained response rates in excess of 80% for clinical associates, dentists, nurses, oral hygienists, pharmacists and physiotherapists. Since baseline, the outright refusals have remained very low at 4.7%. The multiple logistic regression analysis showed that self-identified race was the only significant socio-demographic difference between medical doctor respondents and non-respondents. Black African doctors and Indian doctors were 2.0 and 2.6 times more likely respectively to respond than White doctors (p < 0.05).

**Conclusion:**

Other LMICs can learn from WiSDOM’s lessons of establishing and maintaining a health professional cohort that aims to generate new knowledge for health system transformation.

## Background

Longitudinal cohort studies, where information is collected prospectively, remain critical to the advancement of public health and policy [1]. Their advantages include the ability to identify and relate events to particular exposures; define the presence, timing and chronicity of exposures; establish the sequence of events; eliminate recall bias in participants; and monitor changes over time in cohort members [1–3]. The cohort design has been used in countless studies to determine disease causation [1], examine the determinants of ill-health [4–15], or in population-based studies to assess health outcomes and determinants [3,16–18].

In 2006, the seminal World Health Report underscored the criticality of human resources for health (HRH) and of conducting priority research to overcome the global HRH crisis of under-investment, chronic shortages, maldistribution and poor working conditions [19]. This 2006 report facilitated an increase in HRH or health workforce research of various designs [20–34].

Health workforce cohorts are a subset of longitudinal studies where the sampling population is one or more category of health professionals, the unit of analysis is the individual health professional or worker, and there is an emphasis on repeated measurement of health labour market activities over time [2,35,36]. These activities include information on career preferences, job satisfaction and motivation, location in rural or urban areas, international migration, and the social, demographic, occupational and health system factors that influence these individual preferences or decisions [37,38]. These cohort studies are able to document and monitor the labour market activities of the health workforce over time. Hence, they generate new knowledge to support the design of evidence-informed health policies or interventions and to contribute solutions to address the health workforce crisis [35]. As cohort studies enable the analysis of the degree and direction of change over time [2], researchers can examine the impact of health care reforms and other policy changes on the career choices and job location decisions of the health workforce [39–41]. This impact analysis is essential in light of the global goal of universal health coverage (UHC) and the importance of the health workforce to the achievement of UHC [42].

Health workforce cohort studies are well established in high-income countries [23,25,28,33,39,41,43]. Notwithstanding an encouraging increase in the use of longitudinal study designs in health workforce research in LMICs [21,27,30,32,34,44], prospective cohort studies remain uncommon, especially in sub-Saharan Africa where they are most needed. This is because the HRH crisis is more pronounced in sub-Saharan Africa that suffers from chronic staff shortages and inequitable distribution [45].

South Africa continues to experience significant HRH problems, despite higher national health worker densities than most other African countries, and demonstrable progress in HRH since democracy [46]. South Africa’s HRH crisis is characterised by the maldistribution of health workers, lack of consolidated national health workforce accounts data, fragmented HRH information systems, resource constraints, and insufficient government capacity to guide health workforce planning and strategic management [46,47]. A goal of the 2030 HRH Strategy is ‘data-driven and research-informed health workforce policy, planning, management and investment’ [46]:43. However, achieving this goal will require comprehensive, robust HRH information systems, investment in longitudinal research to analyse health workforce dynamics, including transitions and exits [35], and extensive support from researchers.

Notwithstanding the strengths of the cohort study design, prospective longitudinal studies require long-term investment in research staff, infrastructure and funding for the duration of the study [3,12,48]. The challenges implicit in the cohort study design are incomplete and interrupted follow-up of individuals, attrition with loss to follow-up, and consequent non-respondent bias threatening the representativity of the study population and the conclusions reached [2,49,50]. There is a substantial literature on strategies for cohort retention and maintenance, especially from high-income countries and for epidemiological studies that focus on health outcomes and determinants [3–5,11,12,14]. However, there is a dearth of literature on the methodological considerations and lessons learned from establishing and maintaining health workforce cohort studies, especially in sub-Saharan Africa.

Consequently, the aim of this paper is to address some of these knowledge gaps by detailing the methodology for establishing and maintaining the WiSDOM (Wits longitudinal Study to Determine the Operation of the labour Market among its health professional graduates) health professional cohort study in South Africa. WiSDOM aims to examine the career choices (outcome 1) and job location decisions (outcome 2) of health professional graduates of the University of the Witwatersrand in Johannesburg, South Africa over a period of 15 years [32].

The remainder of this paper is structured as follows: The methods section describes key steps in the establishment of the WiSDOM cohort, and the strategies to maintain and retain the cohort. The results section presents the response rates for the whole cohort and for each of the sub-groups since baseline, the refusal rates, and comparison of socio-demographic characteristics of medical doctor respondents and non-respondents. The discussion and conclusion highlight the lessons learned from the WiSDOM cohort and the value of longitudinal health workforce studies in LMICs.

## Methods

### Establishment of the WiSDOM cohort

**Figure 1.**
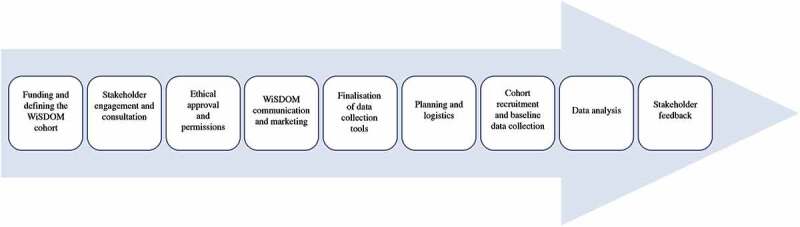
Key steps in the establishment of the WiSDOM cohort

Figure 1 shows the key steps in the establishment of the WiSDOM cohort in South Africa. These steps overlap and in some instances happened simultaneously, but each is described separately for the sake of clarity.

#### Funding and defining the WiSDOM cohort

The award of a South African Research Chair to one of the co-principal investigators enabled the funding and conceptualisation of the WiSDOM cohort.

The cohort consists of the eight professional groups of clinical associates, dentists, doctors, nurses, occupational therapists, oral hygienists, pharmacists and physiotherapists. The rationale for the WiSDOM cohort study is both its scholarly contribution, and its health policy relevance. WiSDOM will generate evidence and new, context-specific knowledge on the South African health workforce and labour market that could inform national health workforce planning and/or projections, influence resource allocation, and inform the design of HRH interventions [32].

#### Stakeholder engagement and consultation

In January 2017, the research team organised an initial consultation workshop to discuss the value and feasibility of the cohort study and to get early inputs from relevant stakeholders, including national government representatives and the various elected student councils to represent the interests of each of the professional groups.

**Figure 2.**
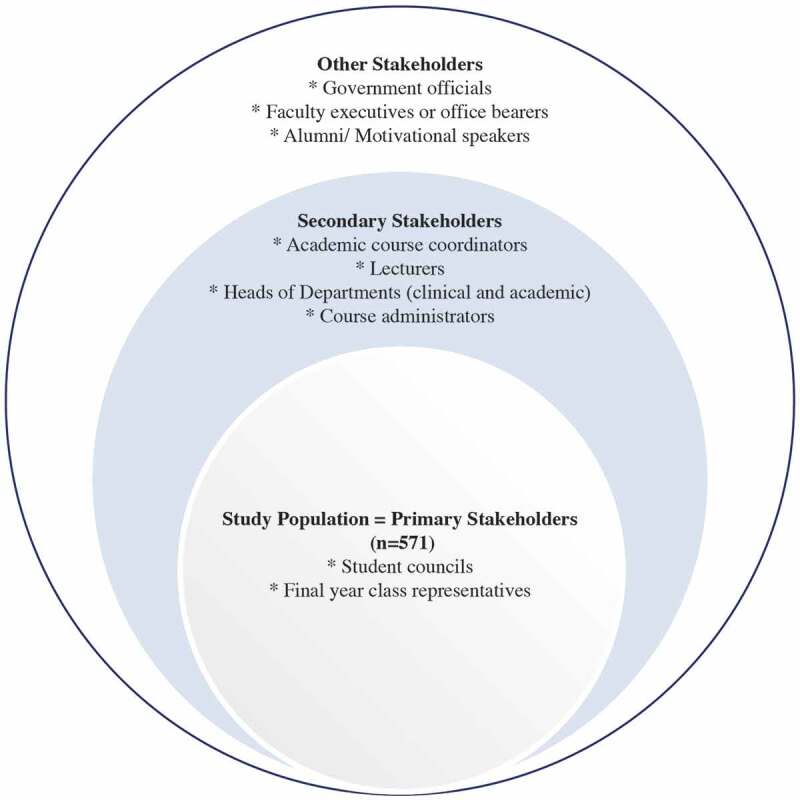
Stakeholders consulted in the WiSDOM health professional cohort study

Between March and May 2017, and prior to the baseline study, we embarked on a further extensive consultative process (Figure 2).

The purpose of the consultation was to obtain stakeholder support for the study, elicit stakeholder inputs on WiSDOM and its longitudinal nature, appropriate communication strategies for different categories of health professional students to maximise voluntary participation, and suitable non-coercive incentives to encourage the participation of the cohort of final year students. The consultation also served as a mechanism to get academic and student support for data collection as part of the academic programme. We also wanted to obtain a schedule of the final year academic programme to enable fieldwork planning.

Our study population or intended cohort was the 571 final year health professional students. They were therefore our primary stakeholders. There were seven elected student councils for the eight professional groups as oral hygiene was combined with dentistry. Each council is the representative organisation of the relevant health professional students. We met with the chairs of each of the seven councils, and in three cases, these chairs were joined by the immediate past chairs (n = 10). Furthermore, each of the professional groups had an elected final year class representative, except medicine with three class representatives. These class representatives communicate with the final year students on various issues (e.g. research, timetables) and act as a conduit for feedback between staff and students. Hence, we met with ten final year class representatives. The 20 students’ inputs shaped the approach to communication with the potential cohort members, the kinds of questions to include in the self-administered questionnaire, the subtle differences to consider across the eight professional groups, and the nature of the incentives.

Our secondary stakeholders (n = 68) consisted of the registrar of the Faculty of Health Sciences (FHS), academics, specifically clinical and academic heads of departments, the academic course coordinators, lecturers in the final year programmes, and course administrators. These individuals were of critical importance to cohort recruitment and baseline study execution, either to provide permission or to facilitate access to students. We met with these individuals and/or made presentations to relevant forums where more than one group were present.

In June 2017, we arranged another consultative workshop with our primary and secondary stakeholders to give feedback on all the individual consultation meetings, and the key issues that emerged, such as recruitment, incentives and long-term retention. We also requested stakeholder inputs on our recommendations for the baseline study execution, and we shared the data collection tools with them. At this June 2017 consultative workshop, one of the occupational therapy students suggested that we prepare a 3-minute video to encourage voluntary participation in the study, in addition to posters and electronic adverts. She recommended that potential cohort members from each of the eight professions should feature in the video, and that the class representatives could distribute the video via social media, mobile phone and/or WhatsApp groups. One of the nursing lecturers referred the research team to an artist that was able to do a low-cost video for R14 000 (~1000 USD).

In the end, we spent around 280 person-hours in the various consultative meetings before the commencement of the data collection for the baseline study.

#### Ethical approval and permissions

We submitted a detailed study protocol, including the draft data collection tools, to the Human Research Ethics Committee (Medical) of the University of the Witwatersrand in Johannesburg, South Africa. We also obtained approval from the relevant university authorities: the Deputy-vice-Chancellor: Academic, the University Deputy Registrar, the Dean of the FHS, the Heads of Schools, and the Heads of Clinical or Academic Departments. In June 2017, we received ethical approval for the study (#M170 550). The ethical conduct of the study is described under data collection.

#### WiSDOM communication and marketing

We created the acronym WiSDOM (Wits longitudinal Study to Determine the Operation of the labour Market among its health professional graduates) both to capture the origins and conceptualisation of the study at Wits University, and the goal of the health professional cohort, namely to generate new knowledge on HRH in South Africa. We then designed an easily recognisable logo, using the university colours, to ensure the unique branding of the cohort study, and to assist with cohort recruitment and retention over time.

The research team contacted the class representatives from each of the health professional groups, and asked volunteers to participate in the marketing video. We wrote a short script on WiSDOM, its aims and why voluntary participation was important. We shared the script with the volunteers, but encouraged them to give their own reasons for participating in the cohort study. Once we had the student actors on board, we contracted the video designer, who worked with this group to do the 3-minute video. Those students who participated expressed both joy and pride in the marketing video, which was widely distributed to all potential cohort members. The video is on the study website https://www.wits.ac.za/wisdom/

We also developed customised adverts and posters to enhance voluntary participation. These materials were distributed through the class representatives, course coordinators, and social media, and were displayed prominently in university venues where students congregate such as the cafeteria and lecture halls.

#### Incentives

During consultation with student councils and final year class representatives on appropriate incentives, the research team received suggestions that ranged from university-branded clothing to unaffordable pieces of equipment or long-term access to medical databases. Additionally, our discussions revealed a common anxiety created by the 2017 changes to the application process for internship (pharmacists and medical doctors) and community service (dentists, nurses, occupational therapists and physiotherapists). Prior to 2017, the application process was manual, and the National Department of Health (NDoH) decided to move to an online system to reduce the manipulation by applicants and urban-bias of placements. However, there was insufficient communication on these changes, and rumours abounded regarding choices and placements.

Hence, the research team decided that a suitable incentive for participation would be a talk on internship or community service by the NDoH after the actual data collection, where all their questions could be answered. We added motivational talks by recent graduates or alumni of the relevant professional groups to share their experiences of internship and/or community service. We included these motivational talks, because clinical associates and oral hygienists do not need to do internship or community service and we wanted to ensure equal treatment of all the groups. We also provided refreshments as part of the NDoH and alumni presentations, and endeavoured to cater for different food preferences, namely kosher, halaal or vegetarian. In addition to the talks and refreshments, we gave each participant an 8GB memory stick with the WiSDOM study logo.

#### Finalisation of data collection tools

The details of the baseline self-administered questionnaire (SAQ) are described elsewhere [32]. Suffice to say that experts from the eight professional groups were asked to comment on the content of the baseline questionnaire, and the tools were tested extensively prior to baseline data collection (Table 1).

**Table 1.** Summary of WiSDOM cohort study methods, 2017–2020

Method	2017 Baseline	2018 Follow-up	2019 Follow-up	2020 Follow-up
**Study population**	All final year health professional students (n=571)	Baseline survey respondents (n=511)	Baseline survey respondents, excluding refusals	Baseline survey respondents excluding refusals
**Recruitment strategies**	Consultative workshop Individual consultations with key stakeholders Customised video, posters and adverts WiSDOM cohort branding	Email to alert cohort members of 2018 survey Circulation of 2 policy briefs	Email to alert cohort members of 2019 survey Circulation of flyer with select 2018 results	Email to alert cohort members of 2020 survey Circulation of newsletter with select 2019 results and reflections from cohort members
**Informed consent andvoluntary participation**	Yes	Yes	Yes	Yes
**Data collection tool**	15-20 minute self-administered questionnaire (SAQ) to obtain detailed baseline information Extensive contact details to enable future follow-up	15-20 minute SAQ on changes since baseline survey Verification of contact details	5-10 minute shortened SAQ on changes since 2018 survey Verification of contact details	5-10 minute shortened SAQ on changes since 2019 and experiences during Covid-19 pandemic Verification of contact details
**Data collection method**	Data collection at Wits Health Sciences Campus in computer laboratory or e-learning room using REDCap Separate data collection sessions for each of 8 professional groups	Web-based survey using REDCap	Web-based survey using REDCap	Web-based survey using REDCap
**Optimising response rates**	Department of Health talk on community service or internship Motivational talk by young, practising health professional Refreshments Opportunity to complete the SAQ online	Weekly email and short message service (SMS) reminders over a period of 8 weeks Follow-up telephone survey by trained external service provider	Weekly email and SMS reminders over a period of 8 weeks Follow-up telephone survey by team members	Weekly email and SMS reminders over a period of 8 weeks Follow-up telephone survey by trained external service provider
**Retention strategies**	Feedback meeting in November 2017 Personalised invitations to feedback meeting	Dedicated website Individual birthday message Communicating results Honorarium of R200 voucher (~13 USD)	Dedicated website Individual birthday message Communicating results Honorarium of R200 voucher (~13 USD)	Dedicated website Individual birthday message Communicating results Honorarium of R200 voucher (~13 USD)

REDCap = Research Electronic Data Capture SAQ=Self-administered questionnaire

#### Planning and logistics

In light of eight professional groups that comprise the WiSDOM cohort study, the study required careful planning and complex logistical arrangements. We obtained the class lists of registered students from the FHS registrar, and the academic class coordinators of the eight professions. We matched each individual on the FHS list with the list obtained from the academic coordinators to check for discrepancies. Once we had verified lists for each health professional group, we created unique study numbers for each group and for each individual cohort member.

The research team developed a detailed project plan, customised for each professional group. We ensured inspection of computer laboratories to determine seating capacity and the functionality of computers. We also ensured the data collection venues were booked at least two weeks in advance. We verified that all computers had access to REDCap (Research Electronic Data Capture) [51].

The team met weekly to monitor progress, and in the week before the data collection started, we met daily. On the day of data collection, the research team arrived at the venue at least an hour before its commencement to ensure that all the logistics were in place, thereby avoiding possible delays and/or technical glitches.

#### Cohort recruitment, data collection and analysis

The cohort recruitment, baseline data collection procedures and analysis are described elsewhere [32], and summarised in Table 1.

At baseline, all participants received a detailed study information sheet and were informed of the longitudinal nature of the study, the voluntary nature of participation, annual follow-ups, and their rights as study participants [32]. The research team is committed and strictly adheres to the Singapore Statement on Research Integrity [52], notably ensuring that participation is voluntary, confidentiality, feedback on study results, and respect for the rights of participants, including their right to refuse study participation without prejudice.

#### Stakeholder feedback

In November 2017, and prior to the end of year graduation ceremonies, the research team invited every cohort member who completed the baseline survey (n = 511), and all relevant stakeholders, including Wits university management, the NDoH presenters on internship or community service, and provincial and national government senior health officials to an evening event. The purpose of the event was to present the preliminary findings from the baseline study, raise awareness among the study participants of the importance of the study, and to encourage long-term retention.

Only ten students attended the 50-person event that included senior government health managers, the Dean of the Faculty, some of the Heads of Schools, and academics. Although the students expressed appreciation for the invitation to the feedback session, many apologised for not being able to attend as it coincided with other celebratory activities such as class graduation parties.

### Maintaining the WiSDOM cohort

**Figure 3.**
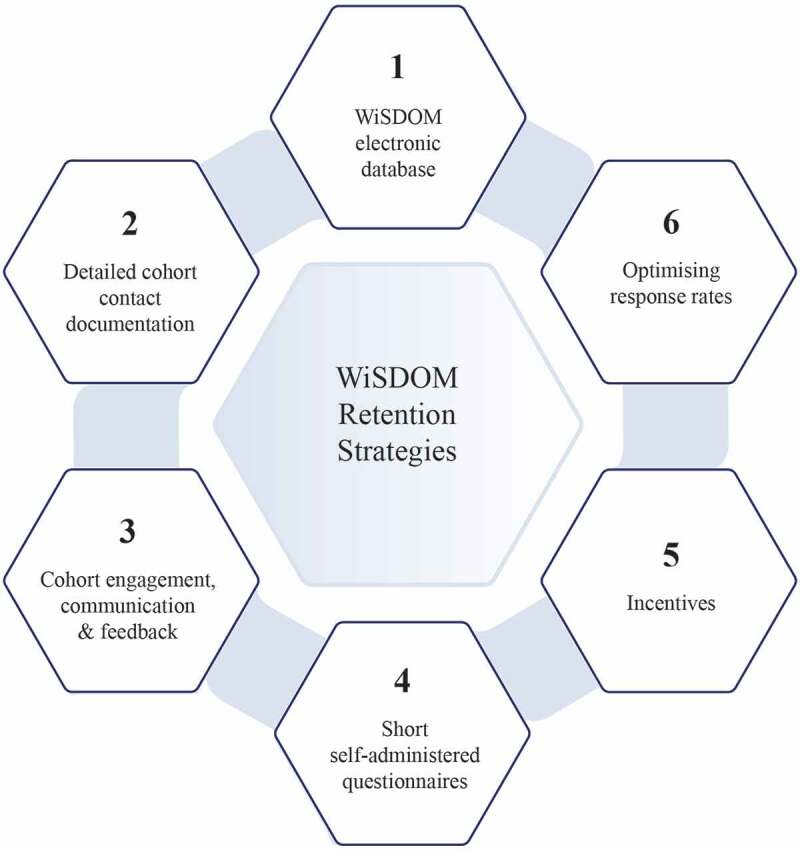
Strategies to maintain and retain the WiSDOM cohort

Given the importance of maintaining cohort participation to prevent selective attrition and to address the long-term study aims, Figure 3 highlights the strategies for maintaining and retaining the WiSDOM cohort. These are inter-related, but each is described separately for the sake of clarity.

#### WiSDOM electronic database

The WiSDOM cohort information is stored securely in REDCap on a Wits University server. REDCap is an online database system, designed to collect, store, secure, organise, and analyse data [51]. This REDCap electronic database allows the research team to access and maintain large volumes of information, as well as regular reviewing and updating of participant contact details.

#### Detailed cohort contact documentation

At baseline, each cohort member provided detailed contact information. This included their own email addresses, physical or postal address, mobile telephone numbers, as well as the details of both parents, siblings and close friends. At each of the follow-up surveys between 2018 and 2020, we requested cohort members to verify their contact details or to indicate any changes. REDCap also allows the research team to keep track of email addresses that have changed between surveys. The processing of the incentive after completion of the survey (see below) also requires cohort members to verify the mobile number for payment.

#### Cohort engagement, communication and feedback

At the beginning of 2018, the research team obtained approval for a dedicated study email address. With the support of the Wits alumni office, we created a WiSDOM database for communication with cohort members, and designed an electronic birthday card to go to cohort members on their birthdays. The team also got support from the Wits communications and marketing division to design a dedicated WiSDOM website https://www.wits.ac.za/wisdom/.

Prior to informing the cohort members of the website, we produced two policy briefs, one that focused on socio-demographic characteristics, and the other that focused on the results of the pro-social choices section of the baseline survey. We chose the policy briefs as our main form of communication for several reasons. Firstly, we wanted to present the key research findings in an accessible format that would appeal to different audiences, including our cohort members and policy-makers. Secondly, the policy brief makes for quick reading, whether for young health professionals at the beginning of their careers, or busy health policy-makers and managers. Lastly, the research team is small, with insufficient capacity to develop customised communication for different audiences.

In July 2018, we informed all cohort members of the new website, the dedicated study email address and the two policy briefs. We asked them for feedback on the website and the policy briefs. We also encouraged them to send any news that they wished to share and informed them of the 2018 survey in the subsequent two to three months. At the end of 2018, we sent them a festive seasons greeting.

In July 2019, we shared soundbites of the results of the 2018 survey, and informed them that we were planning the 2019 follow-up survey.

In 2020, we asked one member from each of the professional groups to share their experiences of the survey and of their working lives in a 50–100 word piece. We also asked them for a head-and neck photograph. We stressed the voluntary nature of the contribution. We combined these narratives with the results of the 2019 survey, and sent out the newsletter in July 2020. We reminded them again that we were planning the 2020 follow-up survey.

#### Short self-administered questionnaires

In 2018, one year after the deadline, we used REDCap to send out the electronic follow-up survey. The survey aimed to obtain detailed information on changing demographics, internship, community service or work experiences, as well as working conditions, and future career and job location intentions. We also requested detailed information on compensation and benefits, with which some of them struggled. We included an open-ended question for comments, either on the survey or on their work experiences as young professionals.

In 2019, the research team took into account the qualitative comments made by cohort members, and decided to focus on those questions that were critical to achieving the long-term goals of WiSDOM. Hence, we shortened the questionnaire considerably to less than 10 minutes. We split the open-ended question into two portions: one dealing with the survey, the other dealing with their working conditions.

Similarly, in 2020, we used the 2019 questions, but added a short section on experiences during the COVID-19 pandemic. The completion time remained under 10 minutes.

At each follow-up survey, we provide a detailed information sheet, we remind all cohort members that participation is voluntary and of their rights. Participants are required to provide consent, via REDCap and can only proceed with the survey after pressing the ‘yes’ button.

#### Incentives

In addition to the strategies described above, every year, we provide each cohort member with an honorarium of R200 (~13 USD) to thank them for participation and to compensate for their data use. This amount is paid as an electronic money voucher within 72 hours following completion of the study.

#### Optimising response rates

For each follow-up survey, we use weekly email and short message service (SMS) reminders for an 8-week period to maximise the response rate. We also sent personalised emails via REDCap to the non-respondents to share the comments of their peers, and to encourage voluntary participation.

In 2018 and 2020, we contracted an external service provider to do telephonic follow-up of the non-respondents. The research team developed a detailed procedure manual to ensure that the service provider conducted the telephone follow-up in a professional and ethical manner. The team spent one-week training the service provider, and doing various dry-runs, including role-plays on how to manage difficult cohort members. We stressed that the service provider staff should encourage the cohort members to complete the questionnaire themselves, and only do a telephone interview if unavoidable.

### WiSDOM data analysis

The data analysis for the baseline survey was described elsewhere [32]. After closure of the 2018, 2019 and 2020 electronic surveys, we imported the data from REDCap into STATA® 14 for analysis. Frequency tabulations were done to calculate the response rates for each of the eight professional groups, as well as the response rate for the overall WiSDOM cohort. We compared the 2018, 2019 and 2020 response rates against the baseline survey response rates in 2017, and between each year of follow-up.

In this study, the response rate refers to the number of a specific health professional group (e.g. dentists) who answered the survey divided by the number of cohort members in that group. Although response rate and completion rate is often used interchangeably, in our study completion rate refers to cohort members that completed all the questions in the survey, and excludes any incomplete questionnaires. Refusal rate refers to those cohort members who refuse to participate further in WiSDOM, which they indicate by opting out of the survey at the consent stage. Non-respondents are individuals who have not responded to the survey, and excludes those who refused participation.

As the greatest non-response rate was for medical doctors, we used bivariate analysis to compare the socio-demographic characteristics of the respondents and non-respondents. We used the Chi-square test to test the difference between these two groups. We constructed a multiple logistics regression model to investigate the independent effects of demographic and socio-economic characteristics (gender, marital status, age, having children, being born in South Africa, profession being first choice and socio-economic status) on the probability of being a medical doctor non-respondent. We calculated odds ratios (OR), 95% confidence intervals (95% CI) and p-values, with those of less than 0.05 considered as statistically significant.

Table 1 summarises the WiSDOM cohort study methods since its inception in 2017.

## Results

### Response rates

We obtained an overall response rate of 89.5% at baseline in 2017. At the first follow-up in 2018, the refusal rate was 3.7%, but the completion rate was 79.6%. Similarly, we had a small refusal rate of 3.9% in 2019, but a 68.3% overall completion rate in that year. In 2020, the cumulative refusal rate was 4.7%, and the overall completion rate increased to 72.8% (Table 2).

**Table 2.** Completion rates in the WiSDOM cohort study methods, 2017–2020

	2017: Baseline (n)	Completion Rates						Total Refusals
		Annual				From Baseline		
		2017: Baseline response (%)	2017 →2018	2018 →2019	2019 →2020	2017 →2019	2017 →2020	
PH	58	95.1%	96.6%	94.6%	98.2%	91.4%	94.8%	1.7%
MD	282	85.7%	66.0%	80.6%	88.6%	53.2%	58.2%	6.7%
DT	17	94.4%	94.1%	100.0%	100.0%	94.1%	94.1%	5.9%
NS	21	95.5%	90.5%	100.0%	100.0%	90.5%	90.5%	0.0%
OT	36	97.3%	94.4%	73.5%	76.5%	69.4%	72.2%	2.8%
PT	46	93.9%	97.8%	88.9%	93.2%	87.0%	89.1%	4.3%
CA	44	93.6%	100.0%	90.9%	100.0%	90.9%	100.0%	0.0%
OH	7	87.5%	100.0%	85.70%	100.0%	85.7%	100.0%	0.0%
TOT	511	89.5%	79.6%	85.7%	91.9%	68.3%	72.8%	4.7%

Table legend: CA = clinical associate; DT = dentist; MD = medical doctor; NS = nurse; OH = oral hygienist; OT = occupational therapist; PH = pharmacist; PT = physiotherapist

The largest decline in completion rates is for medical doctors, among whom we obtained a completion rate of 66.0% in 2018; 53.2% in 2019; and 58.2% in 2020. We also experienced a decline in the completion rates for occupational therapists. Although we obtained a response rate of 94.4% at the first year follow-up in 2018, this declined to 69.4% in 2019, with a slight recovery to 72.2% in 2020. However, for each of the three follow-up surveys, we have obtained response rates in excess of 80% for the six other professions that are part of our cohort. Importantly, we obtained a 100% response rates for clinical associates and oral hygienists in 2018 and again in 2020 (Table 2).

### Comparing medical doctor respondents and non-respondents

Since the baseline survey in 2017, a cumulative total of 6.7% of medical doctors refused to participate in WiSDOM and 35.1% did not complete the survey. Assuming non-completion of the survey as a non-response (n = 118; 41.8%), we have managed to retain 58.2% of medical doctors in the WiSDOM cohort.

Table 3 presents the multiple logistics regression analysis of medical doctor respondents vs non-respondents. Self-identified ‘race’ or ethnic group was the only significant socio-demographic difference between the respondents and the non-respondents. Black African doctors were 2.0 time more likely, and Indian doctors 2.6 times more likely to respond than White doctors. Although female doctors were 1.4 times more likely to be respondents compared to their male counterparts, this was not statistically significant. Married and older medical doctors were less likely to respond to the survey but this was also not statistically significant. The odds of responding were lower for those where medicine was not their first choice (0.4 times), but this was also not significant (Table 3).

**Table 3.** Logistic regression of medical doctor respondents vs non-respondents

Category	Odds Ratio	[95% CI]	P-Value
**Gender**	—		
Male			
Female	1.442	[0.837; 2.484]	0.187
**Marital status**			
Single	—		
Married	0.718	[0.285; 1.803]	0.481
**Age**			
Age	0.968	[0.846; 1.106]	0.631
**Any children**			
No	—		
Yes	0.559	[0.125; 2.491]	0.446
**Born in SA**			
No	—		
Yes	1.375	[0.589; 3.205]	0.461
**Area where born**			
Urban	—		
Rural	0.818	[0.307; 2.176]	0.687
**Race**			
White	—		
Black African	2.011	[1.023; 3.951]	**0.043**
Coloured	0.499	[0.089; 2.795]	0.429
Indian	2.593	[1.163; 5.778]	**0.020**
**Profession 1^st^ choice**			
No	—		
Yes	0.363	[0.146; 0.904]	0.051
**Composite SES**			
Q1 poorest	—		
Q2	0.784	[0.294; 2.086]	0.625
Q3	0.817	[0.286; 2.326]	0.705
Q4	0.843	[0.289; 2.452]	0.754
Q5 richest	0.844	[0.270; 2.635]	0.770

Observations 270

Pseudo R^2^ 0.05

## Discussion

In this paper, we have described the key steps to establish an innovative health professional cohort study at a South African university, the strategies for maintaining the cohort and retaining its members, and the WiSDOM cohort study response rates since inception in 2017.

The strengths of WiSDOM are both its scholarly contribution, and the study’s potential to contribute to HRH policies, and health sector reforms in South Africa. The scholarly contribution of WiSDOM is threefold. Firstly, WiSDOM is a novel prospective health professional cohort study, thus contributing to methodological innovation in the neglected area of HRH research in sub-Saharan Africa. Secondly, the novelty is the simultaneous focus on eight health professional groups in South Africa, which is uncommon in both high-income and LMIC settings. The majority of HRH cohort studies focus on nurses or nurses and doctors [21,23,25,27,28,33,34,39,53]. The Ethiopian cohort study focused on nurses and doctors as these categories tend to dominate frontline health service delivery [30], while the New Zealand’s e-cohort study focused on nurses and midwives [31]. Thirdly, WiSDOM will generate new knowledge on the long-term career choices and job location decisions of health professionals in South Africa. This is already evident from the preliminary study findings on the work status of cohort members with 32.5% of cohort members reporting that they were working in the private health sector in 2020 (https://www.wits.ac.za/wisdom/). Hence the study findings have important implications for HRH planning and policies in South Africa.

Over the four-year period since WiSDOM inception, we have maintained high response rates in excess of 80% for clinical associates, dentists, nurses, oral hygienists, pharmacists and physiotherapists. This is due in large measure to the team’s investment in building relationships with all relevant stakeholders, especially with the student representatives, the engagement and consultation process prior to the baseline study, meticulous planning and preparation, and extensive communication, which laid the foundation for cohort retention. Although the contact tracing differed, the high response rates for the six professions are similar to the response rates of 80% for nurses and 98% for doctors obtained in the Ethiopian cohort study [30]. However, response rates in health workforce cohort studies in high-income countries have ranged from 7.7% to 70% [23,31,53]. In Thailand, which like South Africa is classified as a middle-income country, the Thai Nurse Cohort Study (TNCS) that commenced in 2009, obtained a baseline response rate of 58.6% to the self-administered mailed questionnaire [27]. The second round achieved a follow-up rate of 60.2% [27].

Notwithstanding our success with the six professions and similar strategies to optimise response rates, we have only retained 58.2% of medical doctors, and 72.2% of occupational therapists. We could not find other cohort studies that focus on occupational therapists. Our comparison between medical doctor respondents and non-respondents did not find any statistical significant differences in the majority of social and demographic characteristics. The multiple regression analysis suggests that the controversial issue of self-identified ‘race’ plays a role, with White doctors more likely to be non-respondents. Further research is needed to establish the reasons why this group is less responsive in the annual survey. However, the ‘Medicine in Australia: Balancing Employment and Life (MABEL)’ study that investigates workforce participation patterns and their determinants using a longitudinal survey of Australian doctors obtained an overall response rate of 19.4% at baseline [23]. By the end of the fourth year, 65.4% of the original group of general practitioners and 66.8% of specialists remained in the MABEL survey [49]. Against this background, the WiSDOM response rates for doctors are remarkable. In addition, many other cohort studies have reported follow-up efforts that have spanned across several months [11,30], while a health status cohort study in Thailand pursued non-respondents for a period of 17 months, resulting in a final response rate of 71% [14].

The research team is encouraged by the low percentage of 6.7% of outright refusals among medical doctors. Other researchers have proposed that non-response in a cohort study should be viewed as circumstantial and a temporary problem, unique to each individual, and that perseverance is a key success factor [11]. Hence, the research team needs to deliberate on additional efforts and/or a separate study to determine the reasons for the non-participation of the 35.1% of doctors and 25.0% of occupational therapists.

Our efforts in maintaining communication included the establishment and updating of a dedicated website, sending birthday or festive season messages to cohort members, and annual flyers or newsletters, summarising key aspects of the results. More effort is needed to engage and maintain contact with young, mobile health professionals, and to ensure that they do not suffer from cohort study fatigue. However, the research team is small, consisting of four individuals who contribute 1.2 of a full-time equivalent to WiSDOM, all struggling with numerous competing priorities. Ideally, a dedicated person should be available to design creative communication strategies, such as annual feedback sessions, even if virtual, to cohort members to reassure them that their comments are taken into account, and taken forward (where relevant). Such a dedicated person should also engage with health policy-makers and managers to ensure that the study findings are translated into action, but such a post will require additional funding.

There are several lessons from establishing and maintaining the WiSDOM cohort that may be useful for researchers wishing to embark on health workforce cohort studies in other LMICs. Firstly, dedicated and sustainable funding is needed for these cohort studies. In the case of WiSDOM, an initial five-year research grant from the National Research Foundation enabled the conceptualisation and commencement of WiSDOM. In January 2021, the grant was renewed and increased for a further five years, totalling ten years of committed funding. However, the preferred situation would be for health ministries to develop an essential HRH research agenda and to allocate appropriate research funding. Such an essential HRH research agenda to address priority evidence gaps and concomitant funding would also be in line with the targets set out in the 2030 Global Strategy on HRH [54].

Secondly, HRH cohort studies require extensive stakeholder engagement and participation. The research team spent 280 person-hours of consultation prior to commencement of WiSDOM. Although this was partly due to the complexity of including eight health professional groups in the cohort, the stakeholder engagement, consultation and participation played a central role in realising the high response rates since 2017. Furthermore, the initial investment and ongoing feedback to, and engagement with, various stakeholders are important to build relationships, generate interest in HRH research, and encourage ongoing study support and assistance.

Lastly, health workforce cohort studies are complex undertakings that require meticulous planning for successful execution and retention of cohort members. Although the retention strategies would be country and context-specific, non-negotiable aspects are an updated electronic database, detailed cohort contact information, cohort engagement, communication and feedback, short survey tools, and appropriate incentives.

## Conclusion

The experience with the WiSDOM cohort in South Africa has demonstrated that it is possible to establish and maintain a longitudinal multi-health professional study in an African setting. We hope that our experience will encourage governments and the donor community to recognise the value of HRH research, and invest in longitudinal health workforce cohort studies. Such studies are critical to generate new knowledge for health system transformation, and contribute to the achievement of UHC.

## Data Availability

We cannot share data publicly because this is an active cohort study of health professional graduates. The Human Research Ethics Committee (Medical) of the University of the Witwatersrand has imposed restrictions because of the confidential and sensitive nature of the data. https://www.wits.ac.za/ethics/human-research-ethics-committee-medical.

## Paper context

Within the global context of achieving universal health coverage, health workforce cohort studies can generate new knowledge to support the design of evidence-informed health policies and to contribute solutions to address the health workforce crisis. Yet, these studies remain uncommon, especially in sub-Saharan Africa. This paper details the methodology to establish and maintain the pioneering WiSDOM health professional cohort in South Africa. WiSDOM provides important lessons for health workforce researchers in other low-and middle-income countries.
